# Psychiatric impact of mobile usage on medical student life: Ringxiety, nomophobia, and sleep

**DOI:** 10.1192/j.eurpsy.2021.1059

**Published:** 2021-08-13

**Authors:** L. Shaik, R. Singh, J. Devara, P. Basa, K. Shah

**Affiliations:** 1 Medicine, Mayo Clinic, Rochester, United States of America; 2 Medicine, Metropolitan Hospital, Jaipur, India; 3 Department Of Psychiatry, Griffin Memorial Hospital, Norman, United States of America

**Keywords:** ringxiety, nomophobia, medical students mobile usage, psychiatric effects and sleep disturbance

## Abstract

**Introduction:**

The usage of mobile phones has seen exponential growth worldwide.^1,2^ While college students use mobile applications for educational purposes, the reports of adverse health problems are emerging.^3,4^

**Objectives:**

Investigate the impact of mobile usage patterns on the life of medical students and its association with psychiatric effects concerning ringxiety and nomophobia.

**Methods:**

Data was collected from the 300 medical students of Ashwini Rural Medical College of India through a survey for this cross-sectional study. Chi-square (χ2) was used for statistics that revealed association, mobile phone usage patterns, including time spent before sleep, in classrooms or clinics, and frequency of update checks.

**Results:**

A significant association was found between time spent on mobile before sleep and duration of sleep, and mobile usage in classrooms or clinics and psychological effects (p<0.0001). Significant association observed between mobile use in classes or clinics and the frequency of update checks, and the frequency of update checks and psychological effects (p<0.0001). About 78% of participants distracted in self-study due to mobile. Updates checked every 10 minutes by 14.7%, every hourly by 43%, and during breaks by 42.3%. Mobile low network caused anxiety (13.3%) and irritability (67.3%). About 41.7% of students couldn’t abstain from mobile use for a day. Every student used the mobile phone averagely for 24 minutes before they went to sleep.

**Conclusions:**

Our study results highlight the prevalence of ringxiety and nomophobia in medical school students. With the surging dependency on mobile phones and technology, we need to cautiously monitor its adverse effects on psychology and psychiatric conditions.

